# Effects of Cordycepin in *Cordyceps militaris* during Its Infection to Silkworm Larvae

**DOI:** 10.3390/microorganisms9040681

**Published:** 2021-03-25

**Authors:** Tatsuya Kato, Konomi Nishimura, Ahmad Suparmin, Kazuho Ikeo, Enoch Y. Park

**Affiliations:** 1 Laboratory of Biotechnology, Department of Agriculture, Graduate School of Integrated Science and Technology, Shizuoka University, Shizuoka 422-8529, Japan; konomi971012@gmail.com (K.N.); park.enoch@shizuoka.ac.jp (E.Y.P.); 2 Laboratory of Biotechnology, Department of Bioscience, Graduate School of Science and Technology, Shizuoka University, Shizuoka 422-8529, Japan; suparmin.micro@gmail.com; 3 Laboratory of Biotechnology, Green Chemistry Research Division, Research Institute of Green Science and Technology, Shizuoka University, Shizuoka 422-8529, Japan; 4 Department of Genomics and Evolutionary Biology, National Institute of Genetics, Mishima 411-8510, Japan; kikeo@nig.ac.jp

**Keywords:** *Cordyceps militaris*, cordycepin, entomopathogenic fungi, silkworm larvae

## Abstract

*Cordyceps militaris* produces cordycepin, a secondary metabolite that exhibits numerous bioactive properties. However, cordycepin pharmacology in vivo is not yet understood. In this study, the roles of cordycepin in *C. militaris* during its infection were investigated. After the injection of conidia, *C. militaris* NBRC100741 killed silkworm larvae more rapidly than NBRC103752. At 96 and 120 h, *Cmcns* genes (*Cmcns1*–*4*), which are part of the cordycepin biosynthesis gene cluster, were expressed in fat bodies and cuticles. Thus, cordycepin may be produced in the infection of silkworm larvae. Further, cordycepin enhanced pathogenicity toward silkworm larvae of *Metarhizium anisopliae* and *Beauveria bassiana*, that are also entomopathogenic fungi and do not produce cordycepin. In addition, by RNA-seq analysis, the increased expression of the gene encoding a lipoprotein 30K-8 (Bmlp20, KWMTBOMO11934) and decreased expression of genes encoding cuticular proteins (KWMTBOMO13140, KWMTBOMO13167) and a serine protease inhibitor (serpin29, KWMTBOMO08927) were observed when cordycepin was injected into silkworm larvae. This result suggests that cordycepin may aid the in vivo growth of *C. militaris* in silkworm larvae by the influence of the expression of some genes in silkworm larvae.

## 1. Introduction

The genus Cordyceps is used in traditional Chinese medicine and produces many bioactive compounds that may be useful for treating human maladies [1]. *Cordyceps militaris* is a model fungus in this genus. The species can be induced to produce fruiting bodies on silkworm pupae [2]. This process is used for the industrial production of fruiting bodies in East Asia countries. However, the degeneration of *C. militaris* causes the incomplete formation of fruiting bodies and hinders the development of industrial large-scale production [3].

Cordycepin in the fruiting body of *C. militaris* is a well-known bioactive compound that displays anti-tumor, anti-inflammatory and anti-viral properties [4]. Cordycepin works in various cellular processes, signal transductions [5], apoptosis [6], cell cycle [7] and reactive oxygen species production [8] in mammalian cells. In addition, cordycepin also prevents microorganisms from growing by inhibiting the polyadenylation of mRNAs [4]. Additionally, cordycepin shows insecticidal activity to *Plutella xylostella* (Lepidoptera: Plutellidae) and *Trypanosoma evansi* [9,10]. Large amounts of cordycepin are produced in medium from surface cultures of mycelia [11,12]. This culture method supports large-scale production using mycelial cultivation instead of the preparation of fruiting bodies. More cordycepin is produced in static than in submerged culture. Transcriptomic analysis indicates that hypoxic conditions in static cultivation may be required for the production of cordycepin [13,14]. This result suggests that cordycepin is produced in vivo in the hemocoel of silkworms during the infection.

Cordycepin has many pharmacological and therapeutic activities and has been focused on for the development of drugs. Recently, the physiological role of cordycepin was reported as a suppressor of the immune response in *Galleria mellonella* (Lepidoptera: Pyralidae) [15]. In general, secondary metabolites, such as beauvericin and destruxin produced in emtomopathogenic fungi, are toxic to the immune system of hosts and inhibit the growth of other microorganisms in vivo [16]. In this study, the physiological activity of cordycepin in vivo was addressed by injecting *C. militaris* conidia into silkworm larvae, along with the investigation of morphology during the infection and the investigation of gene expression in *C. militaris* and silkworm larvae.

## 2. Materials and Methods

### 2.1. Microorganisms, Media, and Silkworm

*C. militaris* strains NBRC9787, NBRC100741, NBRC103752, *Metarhizium anisopliae* NBRC8556 and *Beauveria bassiana* NBRC4848 were purchased from the National Institute of Technology and Evaluation in Japan. Fungi were maintained in potato dextrose agar medium. Conidia were obtained as previously reported [17]. Conidia were injected into 5th instar silkworm larvae (Ehime Sansyu, Ehime, Japan) at a defined concentration to induce fruiting bodies. Silkworm larvae were raised on an artificial diet, Silkmate S2 (Nosan, Yokohama, Japan).

### 2.2. Analysis of the Infection

Conidia of each strain were separately injected into silkworm larvae at 1 × 10^6^/mL (25 μL) and incubated at 25 °C. The viability of the silkworm larvae was assessed daily. The effects of cordycepin on the growth of silkworm larvae was investigated by injecting defined concentrations into silkworm larvae together with injection of conidia. 2′-Deoxyadenosine, which is non-toxic, was used as a control instead of cordycepin. Each sample size was described in each figure legend and each experiment was carried out at 3 different times with 3 different batches of larvae. At 72 to 144 h after inoculation with conidia, cuticles, fat bodies and hemolymph were collected, and hyphal bodies and mycelia were assessed microscopically (BX60, OLYMPUS, Tokyo, Japan). Hemolymph was collected by cutting a proleg and fat bodies were collected by scraping off after the dissection of silkworm larvae.

### 2.3. Reverse Transcriptase-Polymerase Chain Reaction (RT-PCR)

Total RNA was extracted from cuticles and fat bodies with Trizol (Thermo Fisher Scientific K. K., Tokyo, Japan). A fat body sample (100 mg) was put into liquid nitrogen and incubated for 1 min. The frozen fat body was crashed and 1 mL of Trizol was added into the sample. After 5 min incubation at room temperature, 200 μL of chloroform was added into the mixture and the mixture was incubated for 3 min. After the centrifugation at 12,000× *g* for 15 min, the supernatant was collected and 2-propanol precipitation was carried out. Precipitated RNA was dissolved with RNA free water, followed by DNase treatment and its purification.

To perform RT-PCR, a PrimeScript RT-PCR Kit (TAKARA Bio, Kusatsu, Japan) used 500 ng of total RNA and 50 ng of cDNA was used for PCR. The sequences of the primers are provided in Table 1. As an internal control, the actin gene (CCM_03787) was adopted to be amplified in parallel with other genes including cordycepin biosynthetic genes (*Cmcns1*–*4*), putative polyketide synthase (CCM_01921) and putative non-ribosomal peptide synthase (CCM_03255).

For RT-qPCR, the THUNDERBIRD SYBR qPCR Mix (TOYOBO, Shiga, Japan) was used after the preparation of cDNA by the PrimeScript RT-PCR Kit. cDNA was prepared using 500 ng of extracted total RNA and 50 ng of cDNA was used for qPCR. Amplified DNA was detected using the Mx3000P system (Stratagene, La Jolla, CA, USA). Data were analyzed by comparative threshold cycle (C_T_) method (2^ΔΔC^_T_ method) using the actin A3 gene (Genbank: NM_001126254.1) as an internal control. Genes including KWMTBOMO08927 (serine protease inhibitor 29), KWMTBOMO11934 (low molecular weight lipoprotein 30K-8), KWMTBOMO13140 (cuticular protein RR-1 motif 42 precursor) and KWMTBOMO13167 (uncharacterized insect cuticle protein) were amplified using primer sets shown in Table 1.

### 2.4. Phenoloxidase Activity

Phenoloxidase (PO) activity was measured as previously described [18]. Briefly, 100 μL of hemolymph was added to a reaction mixture containing 3 g/L l-3,4-dihydroxyphenylalanine (l-DOPA) and 10 mM Tris-HCl (pH 8.0). After incubation for 20 min, dopachrome produced by PO from l-DOPA was recorded by absorbance at 490 nm. Protein concentration was measured using a Pierce BCA Protein Assay Kit (Thermo Fisher Scientific K. K.).

### 2.5. RNA-Sequencing (RNA-Seq)

Total RNA was extracted from the fat bodies of 3 silkworm larvae injected with 100 μL of phosphate-buffered saline (PBS) or 12.5 mM cordycepin, according to 2.3. described above. Injected silkworm larvae were raised for 96 h. cDNA synthesis, strand-specific mRNA library and RNA-seq were performed by Eurofins Genomics K. K. (Tokyo, Japan). In brief, total RNA samples extracted from the fat body of silkworm larvae injected with PBS or cordycepin (*n* = 1 each) were used for strand-specific RNA-Seq library construction using an Illumina HiSeq4000 with the sequence mode 2 × 100 bp. Raw reads were aligned to the reference genome. Gene expression data were calculated by FPKM (fragments per kilobase of exon per million mapped reads) and compared between samples by using the next-generation sequencing (NGS) data analysis system MASER [19].

### 2.6. Statistics Analysis

Statistics analysis was carried out using GraphPad Prism 8 (GraphPad Software, San Diego, CA, USA). The survival rate of silkworm larvae under in each experiment was analyzed using the log-rank test. PO activities and RT-qPCR were analyzed by the unpaired Student’s *t*-test. The difference was assessed with a two-side test.

## 3. Results and Discussion

### 3.1. Viability of Silkworm Larvae after Injection with Conidia

**Figure 1:**
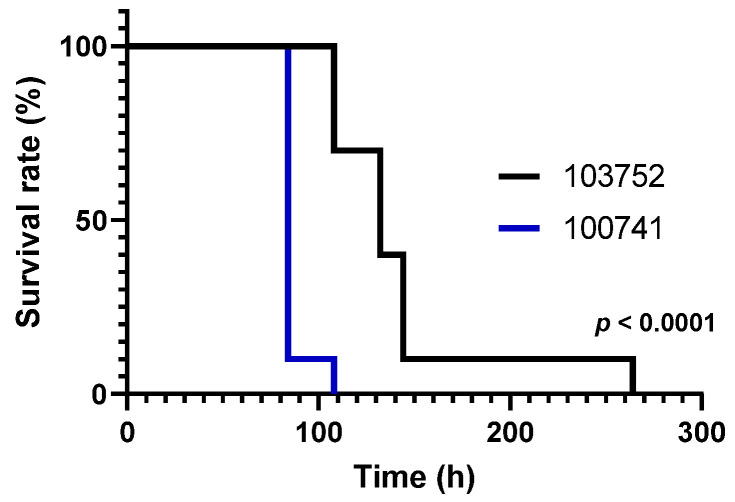
The effect of *C. militaris* conidia injection into silkworm larvae. Black and blue lines indicate *C. militaris* NBRC103752 and NBRC100741, respectively (*n* = 10, triplicate). Results are significantly different using the log-rank test (*p* < 0.0001, χ^2^ = 53.64). PBS was injected into silkworm larvae as a control (data not shown).

First, we investigated the viability of larvae after the injection of conidia of *C. miritaris* NBRC100741 and NBRC103752 (Figure 1). Infection with strain NBRC100741 reduced the viability of silkworm larvae suddenly 84 h after inoculation. All silkworm larvae died by 108 h. In contrast, the viability of silkworm larvae injected with strain NBRC103752 gradually decreased from 108 to 144 h. Dying silkworm larvae turned black. The relative survival of larvae was significantly different using the log-rank test. the median lethal times (LT50s) of strains NBRC100741 and NBRC103752 were 80.8 h (S.E.: 1.069) and 127 h (S.E.: 1.094), respectively. The injection of PBS did not have any influence on the survival of larvae (data not shown). Strain NBRC103752 produced more cordycepin in static culture than strain NBRC100741 [12]. Thus, the amount of cordycepin produced in submerged culture may not be correlated with virulence. A similar result was reported for destruxin produced in *M. anisopliae* where shaking culture is not associated with in vivo pathogenicity to insects [20].

### 3.2. Morphology of C. militaris in Silkworm Larvae during Its Infection

**Figure 2:**
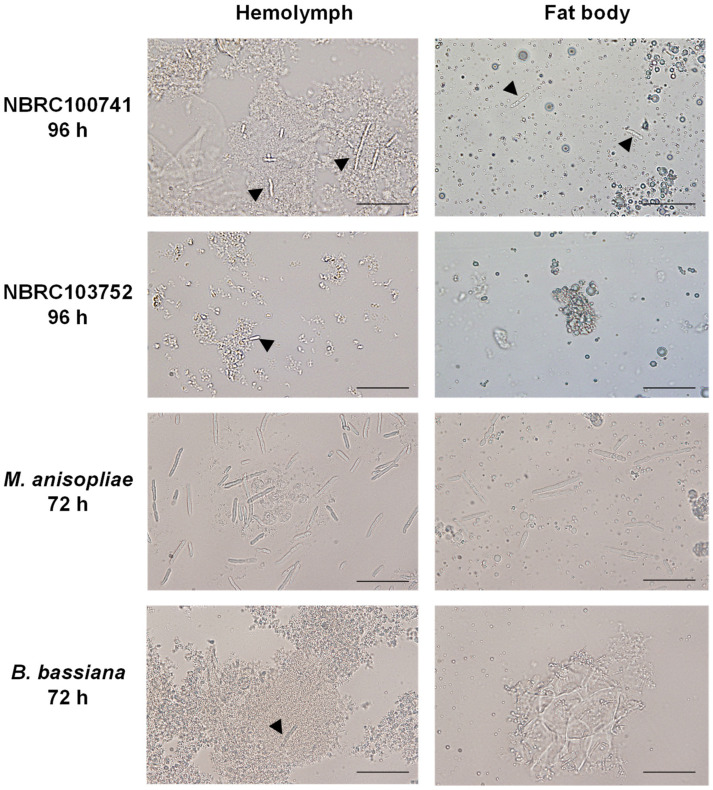
Growth of each entomopathogenic fungi in silkworm larvae. Hemolymph and fat bodies were collected daily after conidia injection and mycelia were observed microscopically. NBRC100741 and NBRC103752 indicate *C. militaris* NBRC100741 and NBRC103752 strains, respectively. Black bars indicate 50 μm. Arrow heads indicate hyphal bodies.

Next, we evaluated the morphology of *C. militaris* in silkworm larvae during the infection (Figure 2 and Figure S1). At 72 h after injection of *C. militaris* NBRC100741, the coagulation of hemocytes appeared in the hemolymph, but no hyphal body or mycelium was observed in the fat bodies or cuticles. After 96 h, some hyphal bodies were observed in the hemolymph and fat bodies, and some mycelia were observed in the cuticles. Colonies of mycelia were observed in the fat bodies and cuticles at 144 h after the conidia infection. In contrast, we observed no coagulation in the hemolymph at 72 h after injection with *C. militaris* NBRC103752 conidia, and hyphal bodies were first observed in the hemolymph at 96 h. Colonies of mycelia were observed at 168 h in the fat bodies and cuticles. These results corroborate our conclusion that *C. militaris* NBRC100741 is more virulent than NBRC103752.

We observed small numbers of hyphal bodies in the hemolymph during the infection of *C. militaris* NBRC100741 as well as *Beauveria bassiana* [21,22], but the number of hyphal bodies were lower compared to *Metarhizium anisopliae* which rapidly grew in the hemolymph (Figure 2). Many hyphal bodies of *Metarhizium rileyi* were also observed in the hemolymph of insect hosts [23]. *C. militaris* infection and growth in silkworm larvae may thus be different from other entomopathogenic fungi.

### 3.3. Cordycepin Biosynthetic Gene Expression of C. militaris in Silkworm Larvae

**Figure 3:**
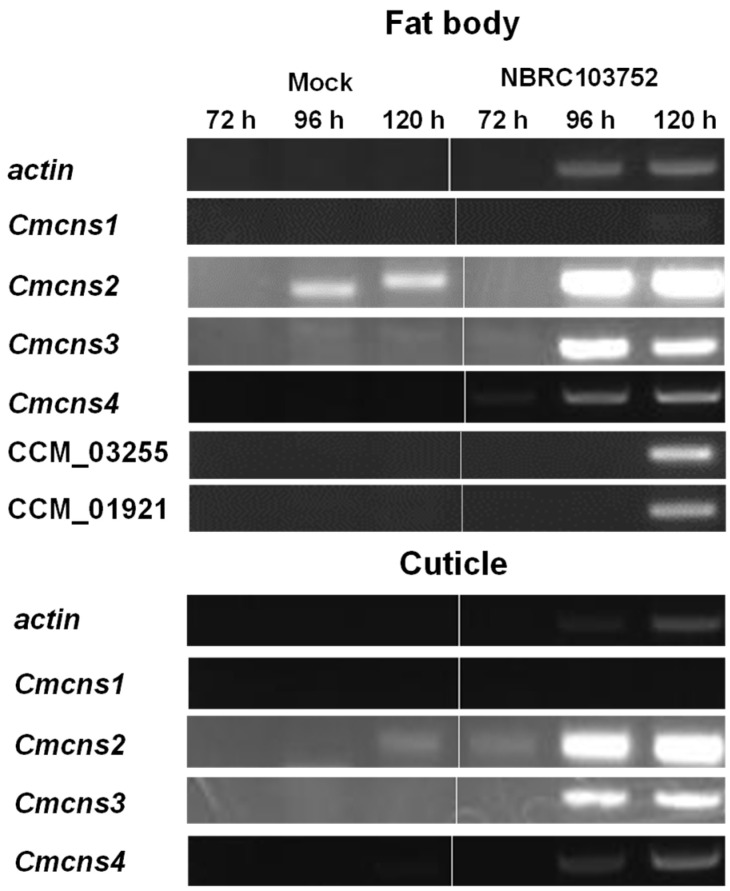
Expression of *Cmcns* genes and genes involved in the synthesis of secondary metabolites in vivo. Silkworm larvae were injected with 25 μL of a suspension of 1 × 10^6^ conidia/mL of *C. militaris* NBRC103752. Fat bodies and cuticles were collected at 3 to 5 days after conidia injection, and total RNA was extracted. RT-PCR was used to assess the expression of each gene. Mock indicates PBS-injection (uninfected control).

*C. militaris* produces cordycepin, which is synthesized by enzymes CmCNS1 and CmCNS2 [24]. CmCNS1 and CmCNS2 likely catalyze the formation of cordycepin from adenosine. Genes encoding CmCNS1 and CmCNS2, which are CCM_04436 and CCM_04436, respectively, are clustered with other genes, *Cmcns3* (CCM_04438) and *Cmcns4* (CCM_04439). We investigated cordycepin production to explore the expression of *Cmcns* cluster genes during infection (Figure 3). In fat bodies, all *Cmcns* genes were upregulated at 96 h and 120 h, along with the actin gene (CCM_03787). This gene expression was concurrent with the decline in larval survival, even though a limited expression of *Cmcns1* gene was observed (Figure 1). Further, the expression of CCM_01921 and CCM_03255, which encode putative polyketide synthase and putative non-ribosomal peptide synthase, respectively, was observed in fat bodies. A protein encoding CCM_01921 displays 43.6% amino acid identity to FSR1 in *Fusarium fujikuroi* IMI58289. The latter is annotated as fusarubin cluster–polyketide synthase [25]. A protein encoding CCM_03255 displays 77.9% amino acid identity to the putative non-ribosomal peptide synthase in *B. bassiana* ARSEF2860 [26]. A protein encoding CCM_01285 shows 72.6% identity with the putative non-ribosomal peptide synthase in *B. bassiana* ARSEF2860 [26], but its expression was not observed in fat bodies (data not shown). Additionally, the expression of CCM_03663 was not observed. This protein is a putative polyketide synthase with 87.6% amino acid identity to putative β-ketoacyl synthase in *B. bassiana* RCEF3172 [27].

### 3.4. Influence of Cordycepin on the Growth of Entomopathogenic Fungi

**Figure 4:**
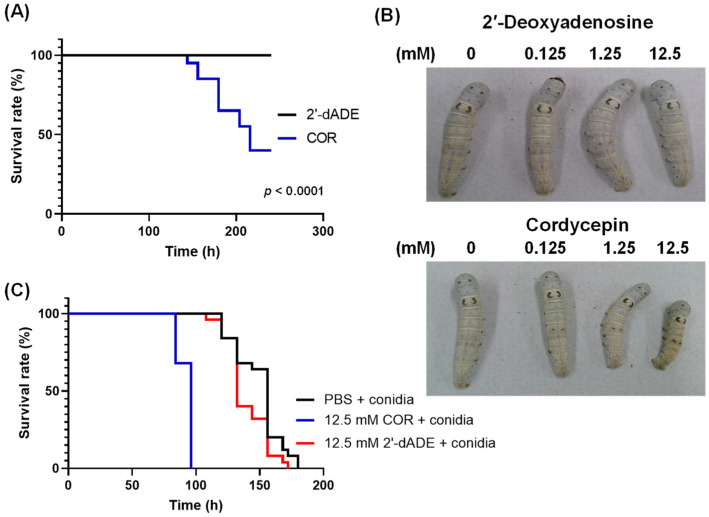
Effect of cordycepin in vivo. (**A**) Survival of silkworm larvae injected with 100 μL of 12.5 mM cordycepin or 2′-deoxyadnosine (*n* = 5, triplicate). COR and 2′-dADE indicate cordycepin and 2′-deoxyadenosine. Results are significantly different using the log-rank test (*p* < 0.0001, χ^2^ = 16.99). (**B**) Appearance of larvae at three days after injection with of cordycepin or 2′-deoxyadnosine. (**C**) Survival of larvae injected with 50 μL of 12.5 mM cordycepin or 2′-deoxyadnosine and 25 μL of 1 × 10^6^ conidia/mL of *C. militaris* NBRC103752 conidia (*n* = 5 or 10, triplicate). COR and 2′-dADE indicate cordycepin and 2′-deoxyadenosine. Survival rates for cordycepin and 2′-deoxyadnosine were significantly different using the log-rank test (*p* < 0.0001, χ^2^ = 83.08).

**Figure 5:**
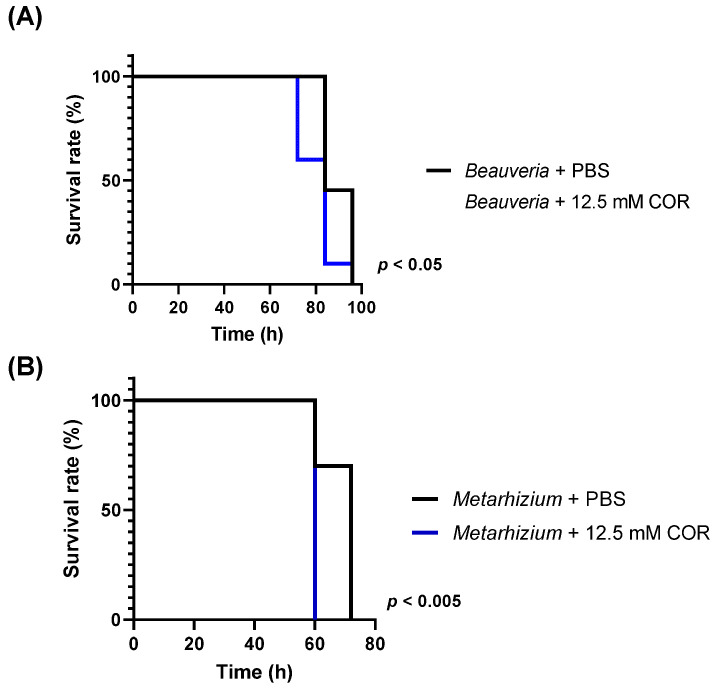
Effect of cordycepin on infection by *B. bassiana* and *M. anisopliae*. (**A**) Survival of silkworm larvae injected with 50 μL of 12.5 mM cordycepin and 25 μL of 1 × 10^6^ conidia/mL of *B. bassiana* NBRC4848 conidia (*n* = 10, triplicate). Results are significantly different using the log-rank test (*p* < 0.05, χ^2^ = 5.766). (**B**) Survival of silkworm larvae injected with 50 μL of 12.5 mM cordycepin and 25 μL of 1 × 10^6^ conidia/mL of *M. anisopliae* NBRC8556 conidia (*n* = 10, triplicate). COR indicates cordycepin. Results are significantly different using the log-rank test (*p* < 0.005, χ^2^ = 10.23).

We injected cordycepin at various concentrations into silkworm larvae (Figure 4A,B). The injection of 100 μL of 12.5 mM cordycepin inhibited normal growth of and killed some silkworm larvae. However, the injection of 2′-deoxyadenosine, which is a non-toxic compound and a precursor of deoxyadenosine triphosphate (dATP), had no influence on the viability (Figure 5A). Further, the injection of cordycepin with conidia accelerated the death of silkworm larvae compared to the injection of conidia with 2′-deoxyadenosine (Figure 4B). Cordycepin appeared to promote *C. militaris* growth in larvae (Figure 4C).

Next, we injected conidia from *B. bassiana* NBRC4848 and *M. anisopliae* NBRC8556 along with cordycepin. *B. bassiana* and *M. anisopliae* are well-known as entomopathogenic fungi, but do not produce cordycepin. Cordycepin significantly increased the virulence of these fungi to larvae (Figure 5). The enhancement of its virulence with cordycepin corresponded to that of *C. militaris* (Figure 4C).

### 3.5. Phenoloxidase Activity in the Hemolymph of Silkworm Larvae Injected with Cordycepin

**Figure 6:**
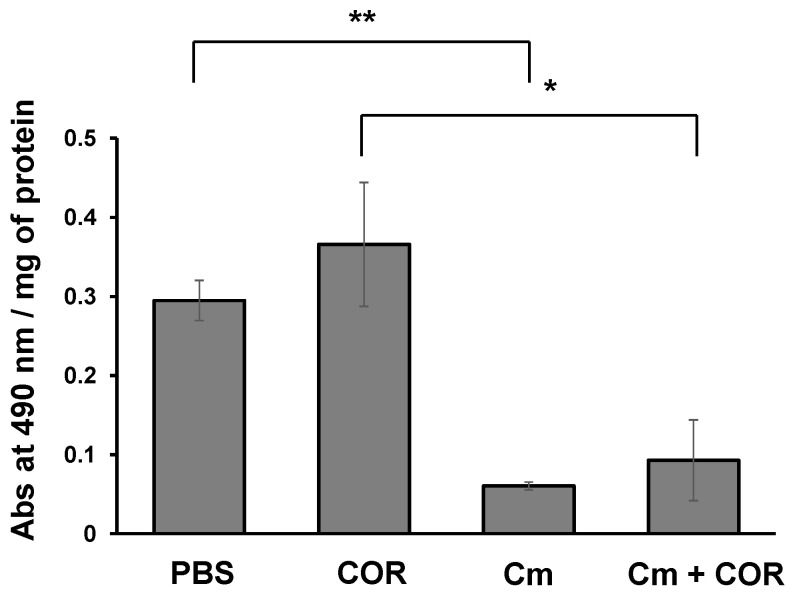
PO activity in hemolymph of silkworm larvae. Assay of PO activity used hemolymph of Scheme 72 h after injection. Cm and COR denote *C. militaris* and cordycepin, respectively. Data were analyzed by an unpaired Student’s *t*-test. The difference was assessed with two-side test. (*n* = 3). * *p* < 0.05, ** *p* < 0.01.

PO activity was measured to investigate the effects of cordycepin on immune response in silkworm larvae. Cordycepin injection did not affect the PO activity in the hemolymph; PO activity in larvae injected with cordycepin was essentially the same as in the control larvae (Figure 6). Additionally, PO activity in larvae injected with conidia was the same as the activity in larvae injected simultaneously with conidia and cordycepin (Figure 6).

### 3.6. The Effects of Cordycepin on the Gene Expression in Silkworm Larvae

**Figure 7:**
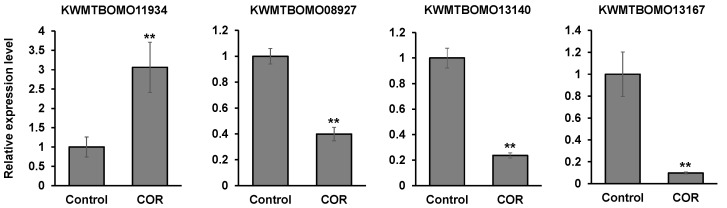
RT-qPCR analysis of each gene shown in Table 2. Total RNA was extracted Figure 3. ** *p* < 0.01.

To investigate the effects of cordycepin on the gene expression in silkworm larvae globally, RNA-seq analysis was performed using RNA extracted from the fat bodies of PBS- and cordycepin-injected silkworm larvae (Tables S1 and S2). The increased expression of a gene (*Bmlp20*, KWMTBOMO11934) encoding a lipoprotein 30K-8 and its isoform was observed in the silkworm fat body when cordycepin was injected into silkworm larvae (Table 2). Additionally, the decreased expression of two genes encoding cuticular proteins (KWMTBOMO13140, KWMTBOMO13167) and a gene encoding a serine protease inhibitor 29 (*serpin29*, KWMTBOMO08927) was also observed. These genes are mainly expressed in the fat body according to the database (https://kaikobase.dna.affrc.go.jp/, accessed on 2 March 2021) and the lipoprotein and the serpin29 gene are in the hemolymph. These results suggested that cordycepin may influence the gene expression in fat bodies, leading to changes in the silkworm physiology. The result of RT-qPCR corresponded to that of RNA-seq analysis (Figure 7).

In a previous study, *Beauveria bassiana* genes, tenellin synthetase (*BbtenS*) and beauvericin synthetase (*BbbeaS*), increased early after the immersion of insect larvae into conidia solution. Expression then gradually declined but increased again at day 12 [28]. Both genes are involved in the synthesis of toxins. A related gene, bassianolide synthetase (*BbbslS*), was expressed at the lower level than *BbtenS* and *BbbeaS*. In the present study, *Cmcns* genes, CCM_01921 and CCM_03255 were observed as silkworm larvae died, but CCM_01285 and CCM_03663 were not expressed. Thus, cordycepin and other secondary metabolites may be important for fungal growth in larvae. Our previous paper shows that the expression of CCM_01285 decreased in aerial and submerged mycelia during cordycepin production in the static cultivation of *C. militaris* [14]. The expression of CCM_01285 may be unrelated to cordycepin production. Some non-specific DNA fragments were amplified using RNA from PBS-injected silkworm larvae and the primer set of the *Cmcns2* gene (Figure 3). We tried to amplify part of the gene with RT-PCR using different primer sets of *Cmcns2*, but some DNA fragments were amplified. We speculate that symbionts, such as yeast-like microorganisms closely related to Cordyceps and Ophiocordyceps in Kermes quercus, may influence the RT-PCR results [29].

Cordycepin accelerated the mortality of silkworm larvae during the infection of *M. anisopliae* and *B. bassiana* in addition to *C. militaris*. Previously, cordycepin was suggested to counter unfavorable conditions caused by the host’s immune system [4]. Kryukov et al. showed that treatment with *C. militaris* enhanced the susceptibility of greater wax moth larvae to *B. bassiana*, and suggested that *C. militaris* uses a different strategy for pathogenesis than *B. bassiana* [30]. This study suggests that cordycepin may be a factor in this strategy and involved in its different infection strategy from other entomopathogenic fungi. Further, cordycepin has insecticidal activity toward *P. xylostella* by oral administration [9]. Therefore, it suggests that cordycepin has some effects on host insects for *C. militaris* to grow easily because, in Figure 4C and Figure 5, cordycepin accelerated the pathogenicity of entomopathogenic fungi.

Recently, it was reported that cordycepin reduced the expression of genes encoding antimicrobial peptides, lysozyme and an insect metalloproteinase inhibitor (IMPI) in *G. mellonella* [15]. In the RNA-seq experiment, the expression of the serpin29 gene was reduced in the fat bodies of silkworm larvae by cordycepin injection (Table 2). A silkworm has 34 serpin genes [31]. Generally, serpins in insects are involved in the protease cascades in hemolymph which activate the Toll pathway and PO activity in the host’s innate immune system [32,33]. In a silkworm, some serpins (1–7, 11–13, 21, 28 and 32) are predicted as a serpin involved in innate immune responses because of its functionality [31]. Figure 6 shows cordycepin does not activate prophenoloxidase involved in innate immunity in silkworms [34]. The decrease in PO activity in hemolymph after injection of *C. militaris* conidia is consistent with a previous study [35]. This prediction corresponds to the results in this study that cordycepin, which reduced the expression of the serpin29 gene, did not have any influence on the PO activity. However, the roles of serpin29 have not been resolved yet.

As well as the serpin29 gene, the expression of two genes encoding cuticular proteins was also reduced by cordycepin injection. The number of genes encoding cuticular proteins covers >1% of the total genes in insects and a silkworm has more than 200 genes encoding cuticular proteins [36,37]. Insect cuticles are composed of chitin and cuticular proteins. Cuticles have many physiological roles in the barrier against the infection of pathogens, the protection from body dehydration, the body injury and insecticides. The reduction of the cuticular protein genes by cordycepin suggests that cordycepin may help *C. militaris* with its growth into cuticles in silkworm larvae.

Recently, it was reported that storage protein Bm30K-19G1 in silkworm hemolymph shows an anti-fungal activity against *B. bassiana* [38]. Additionally, some 30K proteins expressed by pattern recognition molecules are involved in cellular immunity in silkworms by recruiting hemocyte to fungal surface [39]. In this study, the cordycepin injection up-regulated the expression of the gene (*Bmlp20*) encoding a storage protein, 30K-8. The *Bmlp20* gene was slightly expressed in the fifth instar of silkworm larvae and its function is still unknown [40]. It is possible that this storage protein may be involved in the immune system in silkworms even though the expression of this gene was up-regulated by cordycepin.

## Figures and Tables

**Table 1:** Primers used in this study.

Name	Sequence (5′ to 3′)
Cmcns1-F	CATAGTGGGGACGGGATATG
Cmcns1-R	CAAGTGGCTTCTCGCATACA
Cmcns2-F	CCCTGCTCCATGACATTTCT
Cmcns2-R	CAGCGGAAACAGCTCTTCTT
Cmcns3-F	TCCTCAAGCCCACCATCTAC
Cmcns3-R	GCTCCTTGTAGACCGTCTCG
Cmcns4-F	GTATGACGGCCTTGTTTCGT
Cmcns4-R	GCTGAGGACTGCCTCGTAAC
CCM_01921-F	GCAAGACCTTTCGCT TCAAC
CCM_01921-R	CCTTCTCCAAGTTCG TGCTC
CCM_03255-F	ACGGCCACTTGACCTATCAC
CCM_03255-R	ACAATCGTAGCCAACCGTTC
Cmactin-F	GTCCCCGTCATCATGGTATC
Cmactin-R	GGTGTGGTGCCAAATCTTCT
BmactA3_F	AAGCCAACGGAATCCACGAA
BmactA3_R	CTTCATTGTCGATGGGGCGA
KWMTBOMO08927_F	CTCTCCGTCCTGGATTCGTG
KWMTBOMO08927_R	TGTGTTGTGTATGGCCCCTC
KWMTBOMO11934_F	CGGGGAGGGTAAGGAAATCG
KWMTBOMO11934_R	ATCGCCGTATGCGATTCTGT
KWMTBOMO13140_F	CTGATGCCGTCATTCTCCGT
KWMTBOMO13140_R	CGTCAGGCGCAGAGTATTCA
KWMTBOMO13167_F	GGTTGGCGCAGATGGATTTC
KWMTBOMO13167_R	CATATCCCGAGTCTGTGGGC

**Table 2:** Genes differentially expressed in silkworm larvae injected with cordycepin.

Gene	Fold Change *	*p*-Value	Annotation
KWMTBOMO11934	3.87681	0.00005	Low molecular weight lipoprotein 30K-8
KWMTBOMO11934	3.90196	0.00005	Low molecular weight lipoprotein 30K-8 isoform
KWMTBOMO13140	−3.25037	0.0001	Cuticular protein RR-1 motif 42 precursor
KWMTBOMO13167	−4.17149	0.0001	Uncharacterized protein LOC101738360 (Insect cuticle protein)
KWMTBOMO08927	−4.07516	0.00005	Serine protease inhibitor 29 isoform X1

* Fold change indicates log_2_ Fold change. Negative and positive values indicate decreased and increased gene expression by cordycepin injection, respectively.

## Data Availability

The data presented in this study are available on request from the corresponding author.

## Supplementary Materials

The following are available online at https://www.mdpi.com/2076-2607/9/4/681/s1, Figure S1: Growth of C. militaris in vivo. Table S1: Profile of differentially gene expression between the control and cordycepin injection, Table S2: Profile of differentially gene (isoform) expression between the control and cordycepin injection.
