# Supplementary material for: Effects of Cordycepin in Cordyceps militaris during Its Infection to Silkworm Larvae
Source: Microorganisms. 2021 Mar 25;9(4):681. doi: 10.3390/microorganisms9040681 (PMC8065576; doi:10.3390/microorganisms9040681)
Supplement: Supplementary file 1 [file microorganisms-09-00681-s001.zip › Supplementary files/Supplementary Figure S1.docx]

**Figure S1, Kato et al.**

**Figure S1.** Growth of *C. militaris* in vivo. (A) Growth of *C. militaris* NBRC100741 in cuticle and fat body at each time after its conidia injection to silkworm larvae. (B) Growth of *C. militaris* NBRC103752 in cuticle and fat body at 168 h after its conidia injection to silkworm larvae. Black bars indicate 50 μm.
